# Selection of a horizontal reference plane in 3D evaluation: Identifying facial asymmetry and occlusal cant in orthognathic surgery planning

**DOI:** 10.1038/s41598-017-02250-w

**Published:** 2017-05-19

**Authors:** Daniel Lonic, Ali Sundoro, Hsiu-Hsia Lin, Pei-Ju Lin, Lun-Jou Lo

**Affiliations:** 1 Department of Plastic and Reconstructive Surgery, Chang Gung Memorial Hospital, Chang Gung University, Taoyuan, Taiwan; 2 Craniofacial Research Center, Chang Gung Memorial Hospital, Chang Gung University, Taoyuan, Taiwan

## Abstract

Facial asymmetry and dental occlusal cant have been detected in two-dimensional cephalometry using different horizontal reference lines, but equivalent 3-dimensional (3D) reference planes have not been thoroughly investigated. In this study, 3D cone-beam computed tomography scans of 83 consecutive patients were evaluated using a standardized 3D frame and three horizontal reference planes, Supraorbitale (Sor), Frontozygomatic (Z), and Frankfurt horizontal (FH) for cant detection. Canting was defined as a vertical difference between left and right sides of 2 mm or more, and in at least two investigated planes. Concordance for negative canting was found in 38 patients, and for positive canting in 22 patients. Discordance in cant detection was found in 23 patients (28%). 29 patients were found to have canting in at least 2 planes. The FH plane was discordant to the other two planes in 4 patients, the Sor plane in 7 patients and the Z plane in 12 patients. Youden’s index showed the highest performance for FH (0.878), followed by Sor (0.823) and Z plane (0.762). This study revealed that the FH plane was the best method for cant detection in 3D imaging. The FH plane and Sor plane can be combined if orbital asymmetry is suspected.

## Introduction

Facial asymmetry is universally present in the general population and generally not perceived negatively, for asymmetries under 3 mm regarding prominent landmarks such as the oral commissure or the brow level are not clinically noticeable^1, 2^. The underlying skeletal framework shows measurable asymmetries even in highly attractive and balanced faces^3^. Under certain conditions however, symmetry can be unattractive^4^, even though facial attractiveness is determined by balanced facial features, averageness, sexual dimorphisms and youthfulness^5^. Facial asymmetry is a common complaint of maxillofacial patients and can be caused by numerous congenital, developmental or acquired entities^6^. Asymmetry is generally more obvious in the caudal region of the face^7, 8^, mostly due to chin and dental midline deviation, nasal dorsum deviation, occlusal cant, lip cant and orbital dystopia^9^. Patients usually complain about functional and psychosocial problems including perceived facial unattractiveness, malocclusion and altered movement of the temporomandibular joint^10^. Improvement of the facial asymmetry has become as important as correction of the malocclusion in the evaluation and planning for orthognathic surgery. For lower face asymmetry, occlusal canting is undetectable for laypersons when the discrepancy is between 0° and 3°; however, when it exceeds 4°, 90% of the general population will notice the difference^11^.

Different methods for occlusal cant detection have been described. For clinical examination, the angulation of a tongue depressor between the right and left posterior teeth in relation to the inter-pupillary plane can be measured on a frontal photograph^10, 12^. Alternatively, the vertical distance between the maxillary canines and the medial canthi provides similar information^13–15^. However, the patient’s body posture or habitual head tilting during clinical examination may hide asymmetry and mislead the treatment plan^9^.

Two-dimensional (2D) X-ray analysis can detect skeletal facial asymmetry by using vertical lines perpendicular to the horizontal reference line in the antero-posterior cephalogram^16^. Maxillary canting can be detected by measuring the distance between both lateral Orbitale landmarks to the first molar occlusal surface on each side^17–19^. Ricketts assessed occlusal canting by measuring the distance between each maxillary first molar occlusal surface and the inter-frontozygomatic suture reference line^20, 21^. Susarla *et al*. identified occlusal canting by using both Supraorbitale landmarks as a horizontal reference line and measured its distances to both maxillary first molars^14^. The distance between Orbitale and maxillary first molar was measured to identify occlusal canting^22^. However, the accuracy of these 2D quantitative evaluations is limited, because of errors in the patient’s position, 2D image distortion derived from over-projection of anatomic structures, and differential magnification of bilateral landmarks^23^.

Recently three-dimensional (3D) computed tomography (CT) imaging has become more common in evaluating facial asymmetry^24–26^. 3D CT images are ideal for determining size and location of anatomic structures from numerous viewpoints, allowing actual measurements of Euclidian distances and angles^25, 26^. In conjunction with computer-assisted orthognathic surgery planning, 3D imaging allows better prediction, control and accuracy in repositioning and alignment of the jaws^27, 28^. Using 3D imaging, Xia *et al*. detected occlusal canting by defining a triangular plane on the maxillary dentition^29^.

Addressing occlusal canting is one of the most important aspects when orthognathic surgery procedures are planned. The Frankfurt horizontal (FH) plane is a commonly used reference plane to measure and identify maxillary canting^13, 24, 26, 28, 30, 31^. However, there are some issues about the reliability and consistency of landmark identification in 3D images^32^. Certain landmarks were reported to be more reliably identified than others; Schlicher found that the Sella turcica was most consistently and precisely identified, while the right Orbitale was the most imprecise and the right Porion the most inconsistently identified landmark^33^, both being defining landmarks for the FH plane. Especially in difficult facial asymmetry patients with orbital involvement, the use of the FH as a horizontal reference plane may not be suitable to accurately predict the most aesthetically pleasing outcome^34^. In these patients, the use of other horizontal reference planes may be more promising. Therefore, possible occlusal canting has to be assessed from a higher facial plane such as the Supraorbitale (Sor) or Inter-Frontozygomatic suture (Z) plane. The purpose of this study is to investigate which skeletal horizontal reference plane most reliably detects occlusal canting in 3D imaging for consecutive orthognathic surgery patients.

## Results

### Distance from landmarks to the reference planes

**Figure 1:**
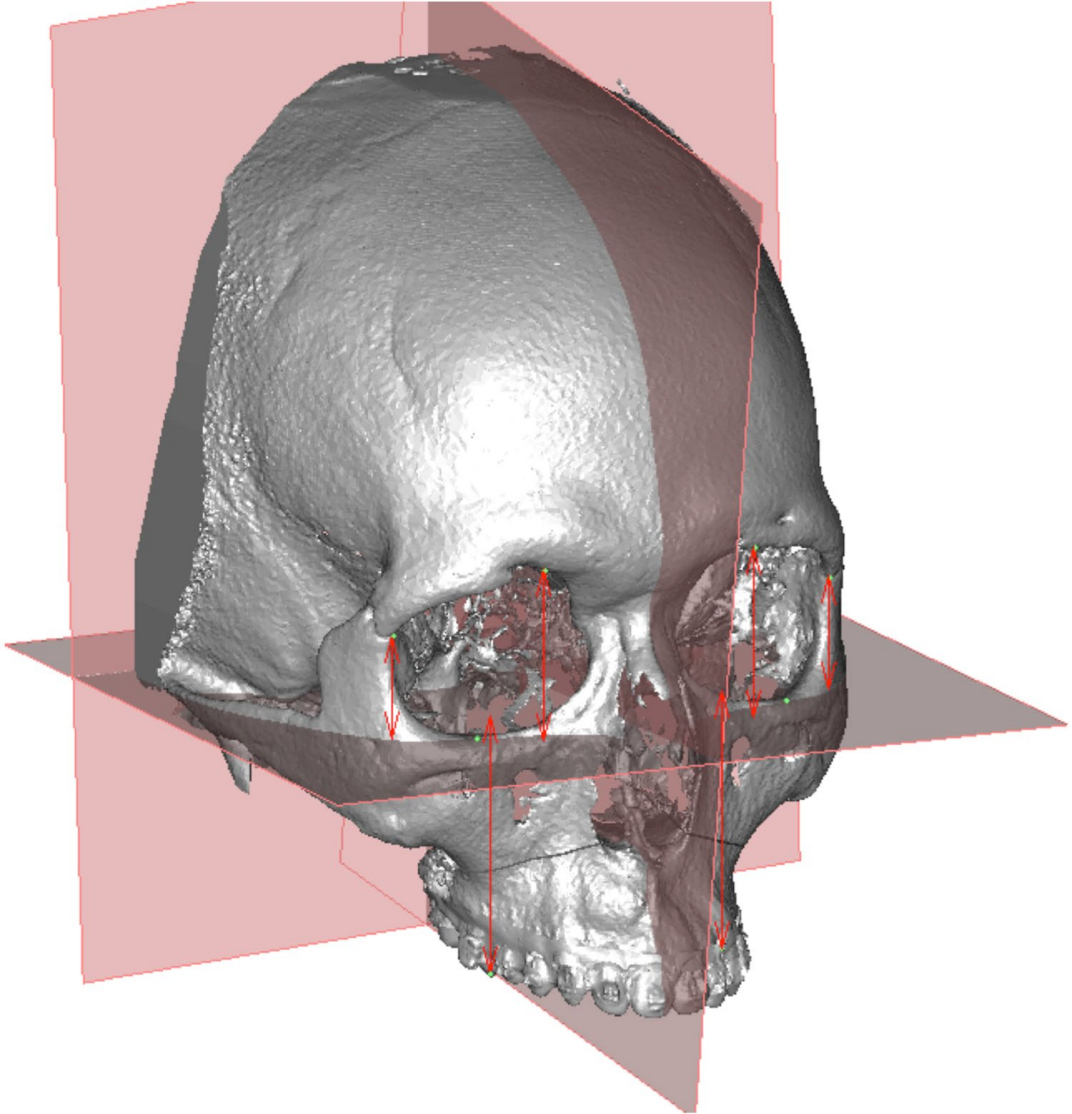
3D reference planes and the measured distances from the landmarks to the FH plane in perpendicular direction.

**Figure 2:**
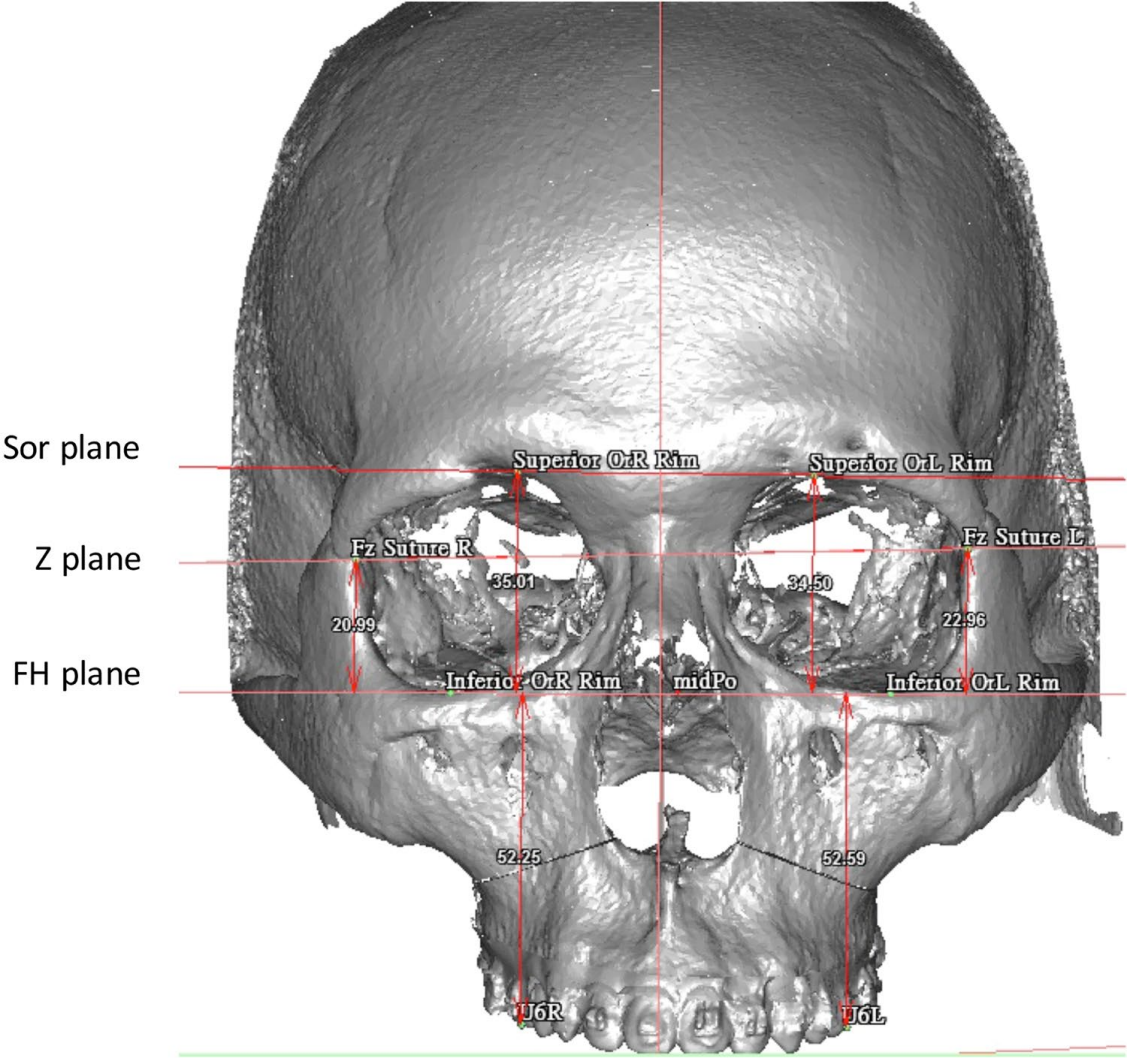
3D image of absolute distance difference calculation. The horizontal reference planes are perpendicular to the coronal plane and therefore appear in a two-dimensional fashion. The distance was measured from each landmark perpendicular to the FH plane. The subsequent distances of the Sor-, Z-, and FH planes were calculated and indicated as (Sor to FH) + (FH toU6) for Sor-U6, (Z to FH) + (FH to U6) for Z-U6, and FH to U6 for both left and right sides to ensure real 3D distance measurement between the planes. The absolute distance differences were calculated as the distance difference between left and right sides for each plane in millimeters.

The 3D frame, landmarks, and three horizontal reference planes were defined (Figs 1 and 2, Table 1). Vertical distances of the 1^st^ molar (U6) to each plane were measured. The difference between the left and right sides was calculated. The mean distance difference for U6-Sor plane was 1.72 ± 1.44 mm, for U6-Z plane 2.01 ± 1.54 mm and for U6-FH plane 1.57 ± 1.48 mm. In the cleft group, mean distance difference for U6-Sor plane was 1.79 ± 1.43 mm, for U6-Z plane 1.99 ± 1.34 mm, and for U6-FH plane 1.71 ± 1.55 mm. In the non-cleft group the mean distance difference for U6-Sor plane was 1.65 ± 1.47 mm, for U6-Z plane 2.04 ± 1.75 mm, and for U6-FH plane 1.42 ± 1.40 mm.

**Table 1:** Definition of landmarks, 3D cephalometric reference planes and absolute distance differences.

Landmark	Definition
**Nasion (N)** ^37^	The midpoint of the frontonasal suture
**Porion (Po)** ^37^	The most superior point of the external acoustic meatus on both sides (Po L, Po R)
**Porion A**	Midpoint between Po L and Po R
**Basion (Ba)** ^37^	The most anterior point of the Foramen magnum
**Supraorbitale (Sor)** ^37^	The most superior point of the superior orbital rim
**Frontozygomatic suture (Z)** ^37^	The most medial and anterior point of each frontozygomatic suture at the level of the lateral orbital rim
**Orbitale (Or)** ^21, 37^	The most inferior point on the inferior orbital rim
**U6** ^37–39^	The most inferior point of the mesiobuccal cusp of each first upper molar in the profile plane.
**Frankfurt Horizontal (FH) plane**	Plane passing through both Orbitale (Or L, Or R) landmarks and the mean of the two Porion (Po A) landmarks
**Sor plane**	Plane passing through both Supraorbitale (Sor L, Sor R) landmarks perpendicular to the coronal plane
**Frontozygomatic (Z) plane**	Plane passing through both Z (Z L, Z R) landmarks perpendicular to the coronal plane
**Sagittal plane**	Plane perpendicular to the FH plane passing through the Nasion (N) and Basion (B) landmarks
**Coronal plane**	Plane perpendicular to both the FH and Sagittal planes passing through the Basion (B) landmark
**Difference Sor(R)–U6(R) and Sor(L)–U6(L) in mm**	Absolute distance difference between both Supraorbitale to FH plane plus U6 to FH plane distances perpendicular to the FH plane on left and right sides
**Difference Z(R)–U6(R) and Z(L)–U6(L) in mm**	Absolute distance difference between both Frontozygomatic suture to FH plane plus U6 to FH plane distances perpendicular to the FH plane on left and right sides
**Difference U6(R)-FH plane and U6(L)-FH plane in mm**	Absolute distance difference between both U6 perpendicular to the FH plane on each side

L: left, R: right.

### Detection of occlusal cant

All three absolute distance differences were concordant for negative cant detection in 38 patients (18 non-cleft, 20 cleft), and concordant for positive cant detection in 22 patients (14 cleft, 8 non-cleft) (Table 2). Subsequently, 23 patients had discordance of planes in cant detection (28%); in these cases, the two concordant planes determined the cant rating. When examining the intra-patient cant detection of the planes in these 23 discordant patients, the FH plane was concordant with one other plane in 19 occasions (11 non-cleft, 8 cleft), the Sor plane in 16 occasions (9 non-cleft, 7 cleft) and the Z plane in 11 patients (6 non-cleft, 5 cleft). Subsequently, the FH plane was discordant to the other two planes in 4 patients (2 non-cleft, 2 cleft), the Sor plane in 7 patients (5 non-cleft, 2 cleft), and the Z plane in 12 patients (4 non-cleft, 8 cleft) (Table 3).

**Table 2:** Patient and cant detection distribution in plane-concordant group (60 patients).

ADD	Cleft	Non-Cleft	Total
**≥2** **mm**	14	8	22
**<2** **mm**	20	18	38
**Total**	34	26	60

ADD: Absolute distance difference.

**Table 3:** Intra-patient cant detection with concordant and discordant patient groups of 23 discordant patients.

Plane	Cleft		Non-Cleft		Total	
	*Concordant with 1 other plane*	*Discordant with other planes*	*Concordant with 1 other plane*	*Discordant with other planes*	*Concordant with 1 other plane*	*Discordant with other planes*
**Sor**	7	2	9	5	16	7
**Z**	5	8	6	4	11	12
**FH**	8	2	11	2	19	4
**Total**	20	12	26	11	46	23

In this table, the total number of concordant planes (*concordant with 1 other plane* columns) is twice the patient number value, because every plane is counted twice in total.

Our model determined that 29 patients (10 non-cleft, 19 cleft) (35%) showed canting with at least 2 mm difference to the U6 level in at least two planes, and equivalently 54 patients (29 non-cleft, 25 cleft) showed no canting with less than 2 mm absolute distance difference to the U6 level in at least two planes (Table 4).

**Table 4:** Cant detection analysis for total patients and cleft/non-cleft subgroups.

	Cant detection	Subjects affected
**Total (n = 83)**	Positive	29
	Negative	54
**Non-cleft (n = 39)**	Positive	10
	Negative	29
**Cleft (n = 44)**	Positive	19
	Negative	25

Chi-Square: *p*-value = 0.0943, no significant difference between cleft and non-cleft groups.

### Comparisons among the three planes

**Figure 3:**
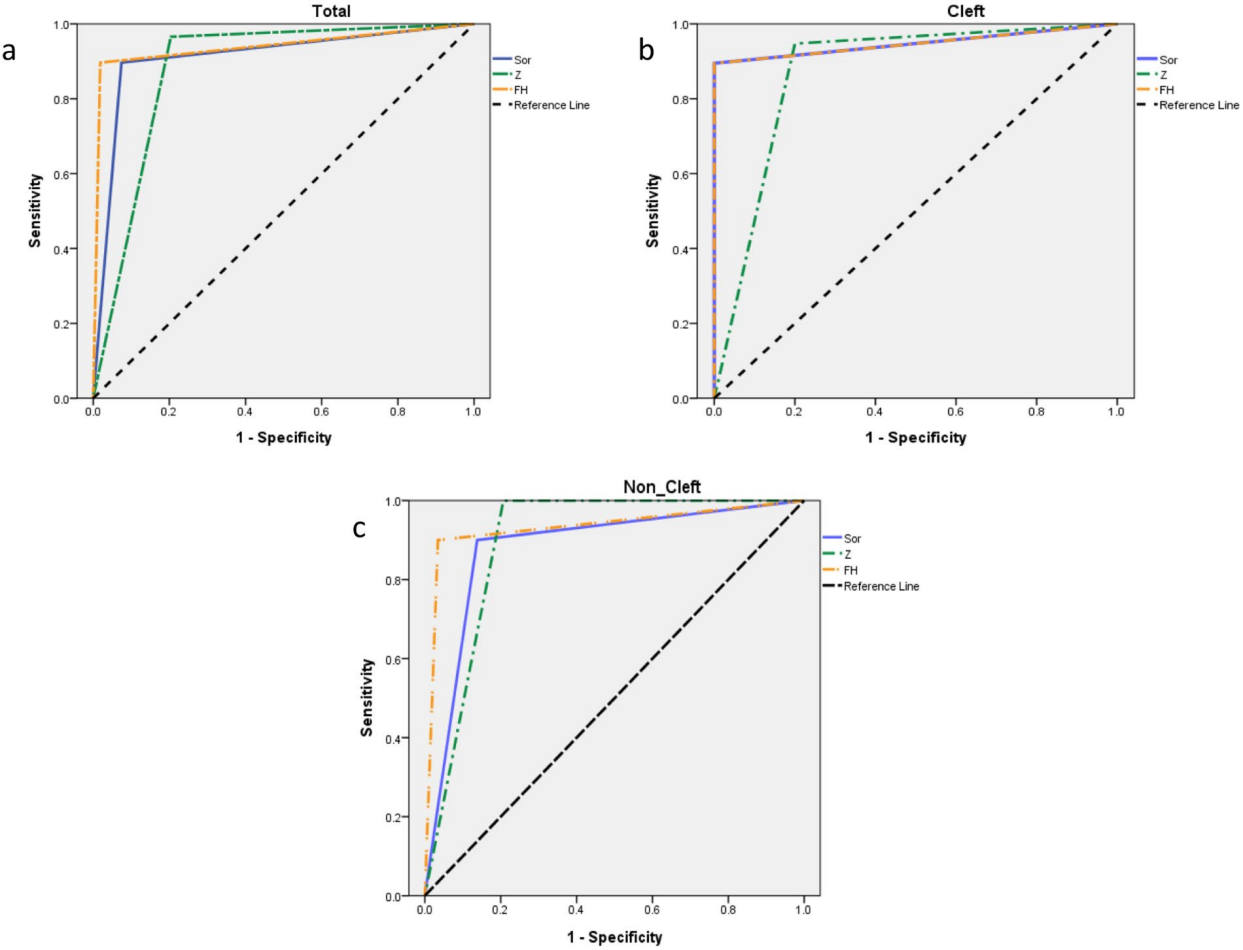
Receiver operating characteristic (ROC) curves for total (**a**), cleft (**b**), and non-cleft (**c**) groups. Diagonal segments are produced by ties.

Sensitivity and Specificity of cant detection for each plane was calculated using the AUC calculation. Youden’s Index for cant detection performance was calculated. All planes showed high overall AUC values with no significant differences between the planes due to overlapping Confidence Intervals (Table 5). For the total patient sample, sensitivity was highest for the Z plane (0.966), followed by both the Sor and FH plane (0.897). Specificity was highest for the FH plane (0.981), followed by the Sor plane (0.926) and the Z plane (0.796). Regarding cant detection performance, Youden’s index was highest for the FH plane (0.878), followed by the Sor plane (0.823) and the Z plane (0.762). For the non-cleft patient sample, sensitivity was highest for the Z plane (1.000), followed by both the Sor and FH planes (0.900); Specificity was highest for the FH plane (0.966), followed by the Sor plane (0.862) and the Z plane (0.793). Youden’s index was highest for the FH plane (0.866), followed by the Z plane (0.793), which was slightly higher than in the Sor plane (0.762). For the cleft patient sample, sensitivity was highest for the Z plane (0.947), followed by both the Sor and FH planes (0.895); specificity was highest for both the FH plane and Sor plane (1.000), followed by the Z plane (0.800). Youden’s index was highest for both FH plane and Sor plane (0.895), followed by the Z plane (0.747). This is also reflected in the subgroup curves of the ROC graphs (Fig. 3).

**Table 5:** Area under the curve (AUC) analysis with Confidence Intervals for all subgroups and sensitivity, specificity and Youden’s Index for all planes.

	Plane	AUC	Sensitivity	Specificity	95% Confidence Interval*		Youden’s Index
					Lower Limit	Upper Limit	
**Total**	*Sor*	0.911	0.897	0.926	0.835	0.987	0.823
	*Z*	0.881	0.966	0.796	0.804	0.958	0.762
	*FH*	0.939	0.897	0.981	0.857	1.000	0.878
**Non-Cleft**	*Sor*	0.881	0.900	0.862	0.729	1.000	0.762
	*Z*	0.897	1.000	0.793	0.799	0.994	0.793
	*FH*	0.933	0.900	0.966	0.000	1.000	0.866
**Cleft**	*Sor*	0.947	0.895	1.000	0.000	1.000	0.895
	*Z*	0.874	0.947	0.800	0.762	0.985	0.747
	*FH*	0.947	0.895	1.000	0.000	1.000	0.895

*No significant difference between planes because of overlapping Confidence Interval.

### Intra-observer reproducibility and inter-observer reliability

For intra-observer evaluation, the mean differences varied from 0.25 to 0.55 mm, and the correlation coefficients (r) were between 0.94 and 0.98 (Table 6), which demonstrated a high degree of correlation between these 2 sets of data. For the inter-observer test, the mean differences in three directions varied from 0.36 to 0.57 mm, and the correlation coefficients (r) were between 0.86 and 0.99 (Table 6), which demonstrated a high degree of correlation between different examiners. The results confirmed satisfactory intra-observer reproducibility and inter-observer reliability in locating landmarks for definition of the reference planes.

**Table 6:** Intra-observer reproducibility and inter-observer reliability of 3D bony landmark identification.

Landmark	Intra-observer reproducibility			Inter-observer reliability		
	Mean	r	p-value	Mean	r	p-value
N	0.43	0.95	0.001*	0.57	0.88	0.003*
Po	0.55	0.98	0.002*	0.38	0.89	0.008*
Porion A	0.30	0.94	0.009*	0.52	0.86	0.002*
Ba	0.25	0.94	0.004*	0.50	0.87	0.003*
Sor	0.41	0.97	0.003*	0.47	0.90	0.001*
Z	0.28	0.98	0.002*	0.44	0.96	0.004*
Or	0.42	0.98	0.006*	0.36	0.99	0.002*
U6	0.33	0.97	0.003*	0.51	0.91	0.008*

r is the Pearson correlation coefficient and *correlation is highly significant at p ≤ 0.05.

## Discussion

In our patient group consisting of 83 patients, we found no statistically significant difference for all planes in terms of sensitivity and specificity, meaning that all of them can generally be used for cant detection. However, when looking at the tendency of Youden’s index in all patients, the FH plane shows the best performance in cant detection; if canting could not be detected by the FH plane, the Sor plane had a better performance in total patients than the Z plane, which showed the highest sensitivity but lowest specificity for canting. When looking at the subgroups, the FH plane showed the best performance for the non-cleft group, followed by the Sor and the Z plane; for the cleft group, FH and Sor plane performed equally well, while the performance for the Z plane was lower. For the non-cleft group however, the Z plane had a slightly better performance than the Sor plane, while the FH plane performed best. The Z plane was higher in sensitivity and lower in specificity than the other two planes, which were higher in specificity but lower in sensitivity, with the exception of the Sor plane being lower in specificity in the non-cleft group (Table 5, Fig. 3a). The FH plane had the highest Youden’s index in all subgroups and total patients. The findings in the total patient group are paralleled by our observations of discordant planes in the 23 ambiguous cases, where only 4 cases of FH discordance were noted, compared to 7 cases of Sor- and 12 cases of Z discordance. In the 4 cases of FH discordance it is interesting to note that there are three cases where the FH plane did not detect canting, while Sor and Z planes detected over 2 mm absolute distance difference between left and right vertical height. In our clinical experience, the detection of cant should be carefully correlated with patient’s facial appearance in surgical planning.

The 3D planning and treatment principal in both cleft and non-cleft patients is the same, and reference planes are needed in this process to ensure precise and adequate surgical planning. The asymmetric deformity is more concentrated in cleft patients; however, it has its focus on the lip and alar region. Although orbital deformity is not a mainstay of cleft lip and palate deformity, it is also detected in this patient population. Therefore, we included a subgroup of cleft lip and palate patients to be analyzed both individually and as a part of the whole group, as we were looking for a plane that can be used in both normal and cleft patients.

As there is a shift from 2D cephalometry to 3D imaging methods for orthognathic surgery planning^35^, reference lines proven to be reliable in 2D^14, 16–20^ have to be reevaluated before using them in a 3D setting. To our knowledge, this is the first study comparing the performance of the 3D FH, Sor, and Z planes for clinical evaluation and surgical planning. Variations of some of these planes have already been investigated for reliability. Oh *et al*. showed that the correlation for clinical cant detection was highest for the 3D FH plane based on either the left or right porion and the 2D frontozygomatic line, and the other reference planes were not put in a reference system, but passed the sella turcica piont. However, our study compares the cant detection performance of true 3D planes derived from the Sor, Z and FH lines known from 2D cephalometry, and evaluates their sensitivity and specificity in both cleft and non-cleft patients.

This study is not without limitations. Although our model seems to reflect our clinical impression of our patients, the patient sample has a problem with statistical significance. The incidence of orbital canting (FH discordance) in our model was 4.8%, so it is hard to collect enough patients to match the McNemar exact test, which shows a calculated sample size of n = 47,027 patients for a power of 0.7; nevertheless, we can use the tendencies of our findings for some suggestions for facial asymmetry evaluation. Although we defined the 2 mm difference as our threshold for the detection of occlusal canting in this study, we can evaluate even smaller amounts of canting when using 3D CT measurements and can address any difference in the occlusal plane when we perform surgical planning and simulation.

When evaluating the patient for canting during computer assisted 3D surgical simulation, one has to be aware that the skeletal finding can differ from the soft tissue relationships^3, 36^. Maxillary canting can have its basis in the upper third of the face, so that the FH plane is not suitable for cant detection and planning. However, even when there is orbital canting in the upper part of the face, it can be compensated by contrary canting of the maxilla so that the soft tissue impression may not even suggest canting. It is important to manage patient expectations if the cause of the facial asymmetry is located in the upper third of the face, because LeFort I osteotomy cannot correct the orbital asymmetry; in these cases, both the Sor plane as well as the Z plane can be helpful in detecting the orbital cant, with slightly higher performance of the Sor plane in total patients and the cleft subgroup.

In conclusion, the FH plane was the best performing investigation method for cant detection. When the cause of the asymmetry is in the upper third of the face, the role of orbital canting in the facial asymmetry can be reliably detected by both Sor and Z planes, while the Sor plane shows more specificity and the Z plane more sensitivity in both cleft and non-cleft patient subgroups. In clinical application, it is suggested to use FH plane first, and add the Sor plane for evaluation if orbital canting is suspected.

## Methods

### Ethics statement

This retrospective study was conducted and approved by the Craniofacial Research Center, Chang Gung Memorial Hospital, Taoyuan, Taiwan. All experiments were performed with the approval of the Institutional Review Board (IRB) of Chang Gung Memorial Hospital (IRB 103-7127B) and the study methods were carried out in accordance with the approved guidelines of IRB. Written informed consents were obtained from the patients or the guardians of the patients younger than 20 years and image release forms for clinical pictures were obtained accordingly for all patients displayed in this publication. Patient’s demographic data included age, gender, diagnosis and operative details. All datasets were retrieved from the center’s databank.

### Patients

The following inclusion criteria for this study were defined: (1) indication for orthognathic surgery for the treatment of dentofacial deformities consisting of both cleft and non-cleft patients, (2) standard preoperative 3D CT scan, (3) surgical planning with Simplant O & O software (Version 17.0, Materialise, Leuven, Belgium), (4) absence of craniofacial syndromes, and (5) absence of pathological head tilting due to cervical spine or muscular anomalies.

Eighty-three consecutive patients (38 male, 45 female) who underwent orthognathic surgery at the Craniofacial Center, Chang Gung Memorial Hospital from 2009 to 2014, with an average age of 21.4 ± 4.8 years (range 15 to 38 years) met the inclusion criteria. All patients were analyzed for the three planes as outlined in this section. Although not all CL/P patients have detectable occlusal canting or facial asymmetry, CL/P patients are associated with individual differences in soft and bony tissue as well as higher degrees of asymmetry compared to the ordinary population. The subjects were therefore divided into 2 subgroups to analyze if there is a difference in occlusal cant detection for the three planes in cleft and non-cleft patients. Group 1 consisted of 44 cleft lip/palate (CL/P) patients (24 male, 20 female) at the average age of 19.2 ± 2.7 years (range 15 to 32 years). Group 2 consisted of 39 non-cleft patients (14 male, 25 female) at the average age of 24.0 ± 5.4 years (range 16 to 38 years).

### Acquisition of cone beam CT scans

A 3D cone beam CT (CBCT) was obtained as a standard acquisition protocol using an i-CAT CBCT scanner (Imaging Sciences International, Hatfield, PA) with a voxel resolution of 0.4 mm. The digital data was exported as a DICOM file and reconstructed to produce a 3D skull model using the Simplant O & O software. The CBCT data evaluation and measurement was performed using the same software.

### Reorientation method and plane definition

To minimize the measurement error, an orientation module was used to standardize the orientation of the image in the FH plane passing the right orbitale (Or R), left orbitale (Or L) and the average porion (Po A) landmarks. The mid-sagittal plane is positioned through the nasion (N) and basion (Ba) landmarks on frontal view and oriented perpendicular to the FH plane. The coronal plane is set up as a perpendicular plane to both FH and mid-sagittal planes passing through the basion (Fig. 1). The four paired hard tissue landmarks (Sor, Z, Or and mesiobuccal cusp of the upper first molar U6) are identified on each side in the CBCT. Using the coronal plane, other two horizontal reference planes are defined, in addition to the FH plane. The Sor plane is defined as the plane perpendicular to the coronal plane passing both left and right Sor landmarks, the Z plane as the plane perpendicular to the coronal plane passing through both left and right Z landmarks. Further definition of the landmarks, planes and distance differences are listed in Table 1.

### Intra-observer reproducibility and inter-observer reliability

10 randomly selected patient samples were taken for validation. The eight previously defined bony landmarks (as shown in Table 1) were marked on the same subjects two times at an interval of 2 weeks from the initial recording. The intra-observer and inter-observer errors were measured using the formula. The Pearson correlation coefficient was determined to validate the intra-observer reproducibility and inter-observer reliability.

$${\boldsymbol{D}}=\sqrt{{({\rm{\Delta }}{\bf{x}})}^{2}+{({\rm{\Delta }}{\bf{y}})}^{2}+{({\rm{\Delta }}{\bf{z}})}^{2}}$$

D being the total error for each landmark, Δx the x coordinates difference, Δy the y coordinates difference and Δz the z coordinates difference on the two measurements [8].

### Measurement of bony landmark distances

Euclidian difference was measured for every investigated plane in every patient. Because the FH plane is the horizontal plane of the reference frame, the vertical distances in millimeters of the U6, Z and Sor landmarks to the FH plane were measured with software integrated tools. These distances were all perpendicular to the FH plane and were measured on both left and right sides. The subsequent distances from U6 to the Sor, Z, and FH planes were calculated and indicated as (Sor to FH) + (FH to U6) for U6 to Sor plane, (Z to FH) + (FH to U6) for U6 to Z plane, and U6 to FH plane for both left and right sides to ensure real 3D distance measurement between the planes. The differences were calculated between left and right sides for each plane in millimeters (Table 1, Fig. 2).

### Model of cant detection between the three planes and statistical analysis

Occlusal cant detection relies on a valid reference plane, and FH-, Z- and Sor planes are the most commonly used. However, orbital asymmetry can distort the analysis for occlusal cant and cause problems in its detection. Therefore we tried to statistically correlate the results of cant detection within the planes to find out which plane has the best performance for occlusal cant detection, even when orbital asymmetry may be present in the patient group. Occlusal canting was defined as distance difference between both sides within one plane being equal to or larger than 2 mm (equivalent to 2° of canting^14^), accounting for not only the general population’s perception, but also the expert threshold on detecting occlusal cant^2^. This occlusal canting had to be detected in at least two out of three of the investigated planes to account for possible orbital asymmetry and subsequent distortion in cant detection. Equivalently, negative canting was noted if the difference was smaller than 2 mm in at least two out of three investigated planes. Chi-squared test was used to note any significant differences between the cleft and non-cleft group for positive and negative canting. For sensitivity and specificity calculation of each plane’s cant detection, every patient was evaluated for plane concordance. Sensitivity and specificity of cant detection for each plane were calculated using AUC calculation. Furthermore, Youden’s index for the performance measurement of each plane was also calculated for all planes for the total patient group as well as for the cleft- and non-cleft subgroups. Data analyses were performed using SPSS Statistic software (version 17, SPSS, Chicago, IL).
